# Assessment of Major Reproductive Disorders in Dairy Cattle in and around Bale Robe, Oromia Regional State, Ethiopia

**DOI:** 10.1155/2021/8855718

**Published:** 2021-01-06

**Authors:** Fedhiko Tolosa, Misrak Netsere, Yitbarek Habtamu

**Affiliations:** ^1^Mekelle University, College of Veterinary Medicine, Mekelle, Ethiopia; ^2^Veterinary Drug and Animal Feed Administration and Control Authority, Addis Ababa, Ethiopia; ^3^National Institute for Control and Eradication of Tsetse Fly and Trypanosomosis, Addis Ababa, Ethiopia

## Abstract

A cross-sectional study by employing a questionnaire survey was conducted to determine the prevalence of major reproductive disorders in dairy cattle and its associated risk factors in and around Bale Robe town from November 2016 to April 2017. Out of 384 dairy cows which were under investigation, 254 (66.15%) had encountered at least one of the reproductive disorders. The major reproductive disorders recorded with high prevalence in the present study included mastitis (20.57%), repeat breeder (17.71%), retained fetal membrane (6.51%), uterine and vaginal prolapse (5.47%), and abortion (4.1%), whereas reproductive problems with lower incidence rate included dystocia, anestrus, hypocalcaemia, uterine discharge, and stillbirth accounting 3.91%, 1.82%, 1.30%, 1.04%, and 0.78%, respectively. The overall incidence of reproductive disorders in this study showed statistical significance (*p* < 0.05) with respect to body condition, age of the animals, production system, and mating system. However, this finding indicated that occurrence of reproductive problems shows statistical insignificance compared to breed, parity, and hygiene of the farm. The prevalence of reproductive disorders in extensive management system (91.02%) was higher than intensive (64.58%) and semi-intensive (57.61%) management systems and also more in local breed (67.93%) than crossbreed (62.29%). However, the prevalence based on parity was higher in primiparous (71.05%) than pluriparous (64.07%) cows. In general, it is recommended that improvement in management system, proper selection of bull and appropriate timing of AI for breeding system, accurate heat detection, routine and periodical examination of cows, balanced feeding, and hygienic condition should be corrected to minimize the incidence of reproductive health disorders and associated risk factors in the study area.

## 1. Introduction

According to CSA, 2015, the country has 56.71 million heads of genetically diverse cattle with 11.38 million milk cows. Livestock production is an integral part of the agricultural activities in Ethiopia engaging 85% of the population directly and indirectly. The livestock sector contributes about 10–12% of the total national Gross Domestic Products (GDP) and 15% of export earnings. Moreover, livestock contributes almost 60–70% of the livelihoods of Ethiopian population by producing a total of 3.07 billion liters of milk annually [1].

Production of livestock has been considered as a major economic sector and still to be continued in the future in most part of the world. Dairy sector development in small holder farming system is one of the key strategic important areas for addressing food security and improved livelihood in developing countries particularly in Ethiopia [2].

Dairy cattle also play a great role in reducing poverty by alleviating economic crisis of the world and generating regular income to the small holder dairy farms [3].

Despite the huge number of cattle population in Ethiopia, the livestock productivity is low due to various constraints like disease, various reproductive disorders, poor nutrition, low genetic potential of indigenous breed, and traditional way of husbandry (management system). These constraints result in poor reproductive performance of dairy cattle and lower economic benefit from the sector [4].

Reproductive efficiency is one of the critical factors for the success of dairy operation, while reproductive inefficiency resulted in inconsiderable economic losses of small holder dairy farm and dairy industry due to prolonged calving interval, early culling of potentially used cows, reduced milk yield and overall production lifetime, and increased cost due to veterinary services [5, 6].

Among the major reproductive problems that have a direct impact on reproductive performance of dairy cows, retained fetal membrane (RFM), repeated breeding (RB), abortion, anestrus, dystocia, uterine discharge, prolapse (uterine and vaginal), mastitis, and stillbirth have been reported to be the most common economic problems [7, 8].

These reproductive problems could also be classified as before gestation (anestrous and RB), during gestation (abortion, vagina prolapse, and dystocia) and after gestation (RFM, hypocalcemia, and uterine and vaginal prolapse). The impaired function of the reproductive system results in failure of a cow to produce a calf yearly and regularly [4, 9].

The biological and economic productivity of livestock production is highly influenced by reproductive performance. In order to improve the reproductive performance, understanding the reproductive disorders has been considered as practical solution. Therefore, the major objective of this study was to assess the prevalence of existing reproductive disorders and determine the associated risk factors of the problem of dairy cattle in the study area.

## 2. Materials and Methods

### 2.1. Description of Study Area

This study was conducted in and around Bale Robe town from November 2016 to April 2017. The town is located in Sinana district of Bale Zone, Oromia Region, and is found 430 km southeast of Addis Ababa, Ethiopia. Bale Zone is characterized by a wide variety of geomorphic landscapes and agroecological zones. Sinana district is one of 18 districts found in Bale Zone. It lays 7° north latitude and 40° east longitude at an altitude of 2270–2690 meters above sea level. Sinana is characterized by bimodal rain fall characteristics. The two seasons are locally called *Bona* and *Ganna*. *Bona* season extends from July to late December and *Ganna* season from mid-March to August. It has an annual rainfall of 1100 mm and the mean annual temperature of 15°C. This bimodal rainfall helps farmers (crop producers) to produce twice a year and livestock producers to get feed twice a year [10].

The agroecology of the district is suitable both for livestock and for crop production. Its land area is approximately 163,854 hectares. It is known for its high production potential for crops such as wheat, barley, beans, and field pea and livestock such as cattle, sheep, goats, horses, and donkeys. The capital city of Bale Zone is also located in this district allowing farmers to get better opportunity to sell their product than in other districts. The livestock population in the district is 445,592 including cattle 287,825, sheep 55,978, goat 15,769, horse 9,200, donkey 14,000, mule 2,820, and poultry 60,000 [11].

### 2.2. Study Animals

A total of 384 dairy cattle, of which local (*n* = 262) and cross (*n* = 122) breeds dairy cow are in the risk of reproductive disorders like abortion, dystocia, repeat breeder, mastitis, still birth, anestrus, retained fetal membrane, uterine discharge, and uterine and vaginal prolapse, were included in this study. These animals were kept in different management systems. Sampled animals constituted different breeds, age, body condition, and parity.

### 2.3. Study Design

#### 2.3.1. Sample Size Determination

The sample size was determined by appropriate sample size determination tools [12] by taking 50% prevalence and 95% confidence interval using the following formula:

(1) $$\begin{array}{c} N=\frac{\left(1.96^{2}\right)\left(P\;\exp\right)\left(1-P\;\exp\right)}{d^{2}}, \end{array}$$

where *N*  is the sample size, *P*exp is the expected prevalence, and *d* is the desire precision.

### 2.4. Sampling Method

A cross-sectional study was conducted on randomly selected local and crossbreed dairy cows (*n* = 384) to find out the prevalence of reproductive disorders. The detailed history of the cow including breed, age, body condition, parity, the previous Artificial Insemination (AI) or natural mating, and management system was obtained from the cattle owners through structured questionnaire format.

### 2.5. Data Management and Analysis

The collected data were recorded in Microsoft Excel spread sheet and coded properly. The data was presented using the descriptive statistics and analyzed using a software SPSS® version 20. Different factors including age, breed, parity, management, and body condition score that were considered during the study period were analyzed using the chi-square technique. In all chi-square test application, probability of *p* < 0.05 was considered statistically significant.

## 3. Results

### 3.1. Socioeconomic Characteristics of the Respondents

The result indicated that the majority of the respondents were male (57.55%) and the remaining 42.45% were female. From the total of 384 respondents, the percentages of various age groups of 10–20, 21–30, 31–40, 41–50, and above 51 years were 00.00, 29.43, 47.14, 19.79, and 3.65 percent, respectively. This result showed that most of the people actively engaged in dairy activities were in the productive age. The educational level of the respondents involved in dairy cattle rearing in the study area was diverse from illiterate (34.8%) to literate people in that they do have diploma and above (18.22%), secondary education (15.93%), and primary education (31.07%).

Majority of the respondents were involved in crop and livestock production systems (67.45%) and the remaining 32.55% engaged in livestock production alone. From the total of 384 dairy cattle, 96 (25%) were managed under intensive production system, whereas 210 (54.69%) and 78 (20.31%) were semi-intensive and extensive production system, respectively. The size of the dairy farms varies from small size (68.75%) to the large size farms (5.21%), while 26.04% of the respondents were medium size dairy farms. The sources of income of 10.94 and 5.47 percent respondents were salary and other business activities (briefly described in Table 1).

### 3.2. Animal Husbandry Activities of the Respondents

According to information obtained, from the total of study animals, 262 (68.23%) were local breed and the rest 122 (31.77%) were crossbreed. Most of the animals belong to the age group of >7 years totaling 146 animals (38.02%); the rest of them were 52 (13.54%), 58 (15.10%), and 128 (33.33%) belonging to the age group of <3 years, 3–5 years, and 5–7 years, respectively. The parity of the animal was categorized as primiparous and pluriparous, which, in numbers, showed 114 (29.68%) and 270 (70.31%), respectively.

Among the study animals, 158 (41.15%) and 99 (25.78%) were breed by AI and NS, respectively, whereas 127 animals (33.07%) were bred by both AI and natural mating by bulls. According to the body condition of the study animals, the majority of them were poor body condition (46.61) and only 8.85% of animals were good body condition. The remaining 44.61% of animals come under the category of medium body condition.

The following table shows the frequency and percentages of breed of study animals, age of animal, parity, mating system, and body condition (Table 2).

### 3.3. Major Reproductive Disorders Identified

The present study revealed that, from a total of 384 animals examined, 66.15% (*n* = 254) were affected by either one or more reproductive disorders (Table 3).

The major reproductive disorders identified in the study area were dystocia (*n* = 15 (3.91%)), abortion (*n* = 16 (4.17%)), clinical mastitis (*n* = 79 (20.57%)), hypocalcemia (*n* = 5 (1.30)), RFM (*n* = 25 (6.51%)), RB (*n* = 68 (17.71%)), anestrus (*n* = 7 (1.82%)), uterine and vaginal prolapse (*n* = 21 (5.47%)), uterine discharge (*n* = 4 (1.04%)), still birth (*n* = 3 (0.78%)), and mixed disorders (*n* = 11 (2.86%)) as summarized in Table 4.

### 3.4. Associated Risk Factors with Reproductive Health Problems of Dairy Cattle

In this study, among the associated risk factors, body condition, production system, hygienic condition of the farm, age, parity, and mating system were considered to assess its association with the occurrence of the reproductive problems (Table 5).

In the present study, no statistically detectable effect (*p* > 0.05) of breed was shown on the cumulative incidence of major reproductive health problems evaluated. However, the incidence of the reproductive problems was higher in local cows (67.93%) than in cross cows (62.29%) (Table 5). Breed had statistically significant effect on hypocalcemia and mixed disorders. No statistically significant difference was observed in cases of dystocia, abortion, mastitis, RFM, RB, anestrus, uterine and vaginal prolapse, uterine discharge, and stillbirth (Table 6). The incidence of hypocalcemia was significantly higher in crossbred cattle (3.28%) than in local breed (0.38%) and the incidence of mixed disorders in local breed cows was higher than crossbreed cows (Table 6).

Prevalence of reproductive disorders in primiparous and pluriparous cows was 71.05% and 64.07%, respectively. Parity had no significant effect (*p* > 0.05) on the overall prevalence of problems (Table 5). However, parity had statistical significance (*p* < 0.05) with respect to dystocia with higher prevalence in primiparous cows (8.77%) than 1.85% in pluriparous cows (Table 7).

The prevalence of reproductive disorders in poor, medium, and good body conditions animals had no significant effect (*p* > 0.05) on the overall prevalence of problems (Table 5). However, the body condition of the animals was statistically significant (*p* < 0.05) with respect of dystocia with higher prevalence in poor body condition (11.48%) than medium (2.69%) and good (1.59%) body condition animals, respectively (Table 8).

There was higher prevalence of dystocia (11.54) in the age group of less than 3 years than 3–5 years (6.90%), 5–7 years (00.00), and >7 years (3.42%), whereas the percentage of abortion was high in older cows above 7 years (8.22%) than other age groups (Table 9).

The prevalence of general reproductive disorders in extensive management system (91.02%) was higher than that in intensive management system (64.58%) and semi-intensive system (57.61%). This result showed that management system at which the owners maintained their animals revealed statistical significance (*p* < 0.05). But the result in this study indicated that the reproductive in relation to hygiene condition of the farm was 69.83% in poor hygiene condition, 64.91% in medium hygienic condition, and 52.94% in good hygienic condition of the farm. There was no statistically significant (*p* > 0.05) variation in prevalence of total reproductive health problems among the studied hygienic conditions of the farm group (Table 5).

## 4. Discussion

Out of the total examined dairy cows, 66.15% (*n* = 254) were found to be affected with at least one of the reproductive health problems, which means the overall finding showed that nearly two of three cows were observed with clinically manifested reproductive disorders. The prevalence of major reproductive disorders reported in this study is in agreement with prevalence of 67.7% reported by Haile et al. [13]. The prevalence in this study is higher than that of studies conducted by Haile et al. [8], Dawit and Ahmed [14], and Ayana and Gudeta [15] who reported overall prevalence of 43.07%, 40.25%, and 35.2% of major reproductive problems, respectively. However, the incidence of the reproductive problems in this study is lower than that of study conducted by Alemselam et al. [16] and Tibletse [17] who reported that the prevalence was 91.2% and 73.7%, respectively. This variation in prevalence may be due to environmental factors, breed of animals, parameters included in this study, sample size, and variation in management system that is applied to different dairy farms.

In the present study, no statistically detectable effect (*p* > 0.05) of breed was shown on the cumulative incidence of major reproductive health problems evaluated. However, the incidence of the reproductive problems is slightly higher in local cows (67.93%) than in cross cows (62.29%). The analysis could not detect significant effect of breed on occurrence of reproductive health problems. The lower incidence of the reproductive problems in crossbreed cows is suggestive of better care, with better feeding and health care than the indigenous cattle.

The incidences of reproductive disorders in primiparous and pluriparous cows were 71.05% and 64.07%, respectively, and parity had no significant effect (*p* > 0.05) on the incidence of the reproductive problems. This finding is in agreement with the previous work in Ethiopia by Ayana and Gudeta [15] who did not detect the influence of parity on the incidence of reproductive problems in dairy cows. However, the slightly high incidence of reproductive problems in primiparous cows might be due to the factors that heifers are highly prone for dystocia and other related reproductive problems.

The incidence of reproductive problems were higher in cows that were bred by NS (80.8%) than those that were bred by AI (63.28%) and both (58.26%). The high incidence of reproductive problems in cows bred by NS might be the effect of transmission of venereal disease, which may lead to endometritis, RB, stillbirth, abortion, etc. In AI, better care was taken at semen processing centers to produce good quality semen and the incidence is further low in both AI and NS mating systems because of better performance of bulls to detect the estrous cycle for better conception rate avoiding RB, anestrus, etc. The wide variation among the three mating systems might be due to the literacy of the cattle owners. In contrast to this study, Ayana and Gudeta [15] stated that the reproductive problems were higher in cows that were bred by AI than those that were bred by NS. This could have been due to the size and sex of the calf and breeding season of the year.

Body condition score of the study animals did influence the incidence of reproductive health problems in the present study; incidence is higher in cows with poor body condition (68.85%) than medium (65.76%) and good (65.07%) body conditions. The reproductive health problems showed significant variation with regard to body condition score. This is in agreement with the report by Ayana and Gudeta [15] who report 44.7% and 35.4% in poor and good body condition. However, Wujira and Nibret [18] stated that the prevalence of the major reproductive disorders decreased from poor to medium body condition with the prevalence of 30.8% and 4.8%, respectively. This variation in the occurrence of reproductive problems in various body conditions may be due to sample size, production system, literacy of the farmers, and breed as well as environmental factors.

In this study, clinical mastitis, RB, RFM, uterine and vaginal prolapse, and abortion were found to be the major reproductive health problems containing 20.57%, 17.71%, 6.51%, 5.47%, and 4.17%, respectively. Other reproductive disorders observed with lower prevalence included dystocia, mixed disorders, anestrus, hypocalcaemia, uterine discharge, and stillbirth accounting for 3.91%, 2.86%, 1.8%, 1.30%, 1.04%, and 0.78%, respectively.

The high prevalence of clinical mastitis observed from all study cows was 20.57%. This result has fairly agreed with the prevalence of 20.4% reported by Fasil et al. [19]. However, the result of the present study was lower than findings of prevalence of 26.5% reported by Lakew [20]. Variation in occurrence of mastitis may be due to variation in the hygienic condition of the house and milking procedure and also variation in the burden of pathogen on different environmental settings.

The prevalence of repeat breeder in present study was 17.71% which is slightly less than findings of prevalence of 21.8% and 21.0% reported by Hunduma [21] and Alemselam et al. [16] and higher than the incidence reported by Getachew and Nibret [22] and Haile et al. [13] with the prevalence of 15.9% and 6.2%. High incidence of repeat breeding could be due to lack of nutrition, improper insemination and timing of AI, and poor semen quality at the end point.

The prevalence rate of RFM of 6.51% obtained in a recent study is similar to the prevalence of 7.18% reported by Haile et al. [8] and lower than those of 19.2% by Gashaw et al. [23] and 10% by Fasil et al. [19]. The variation in the incidence of RFM may be due to predisposing factors, including nutritional status of animals and management system.

The prevalence of uterine and vaginal prolapse of 5.47% obtained in present study is in agreement with the prevalence reported by Kidusan [24] as 5.2%. But it is higher than the prevalence of 0.43% and 3.44% reported by Dawit and Ahmed [14] and Haile et al. [8], respectively. This variation happened as a result of incidence of dystocia and associated risk factors.

The prevalence rate of abortion obtained in this study was 4.17% which was slightly less than findings by Getachew and Nibret [22] who reported that the abortion rate was 5.3% and higher than the prevalence rate reported by Gizaw et al. [25] as 2.23% and Gashaw et al. [23] as 1%. These results suggest that breed geographic location and procedural difference are all sources of differences in prevalence of abortion.

In the present study, the incidence of dystocia of 3.91% is in agreement with the findings of Gashaw et al. [23] who reported that the prevalence of dystocia was 3.8%, but was higher than the 2.9% prevalence reported by Hadush et al. [7]. However, the current finding is lower than the prevalence of 8.7% and 7.93% by Kidusan [24] and Simret [26], respectively. Age of animals, breed, and parity are all factors causing variation in occurrence of dystocia. Inseminating cows with semen collected from large sized bulls and naturally mating a heifer with large sized bulls without taking into consideration the size and age of cows are an important precipitating factor for dystocia.

Anestrus is the other important reproductive disorder obtained in the present study with the prevalence rate of 1.82% similar to a report that found 1.7% by Bitew and Shiv [27]. However, the prevalence in this study is lower than the prevalence value of 12.26% documented by Haile et al. [8]. These may be due to variation in management and breed of the animals as well as environmental factor.

The prevalence of stillbirth, hypocalcemia, and uterine discharge was 0.78%, 1.30%, and 1.04%, respectively. The prevalence of stillbirth is in agreement with the prevalence of 3.01% reported by Dawit and Ahmed [14] and the prevalence of uterine discharge fairly agreed with prevalence of 1.2% reported by Getachew and Nibret [22].

The prevalence rate of hypocalcaemia of 1.30% recorded in this study is much lower than the result of 17.5% reported by Fasil et al. [19]. This difference may be management, type of breed, and study population. Most of the literatures suggest that when the incidence of milk fevers increases above 10% in their third or latter lactation, considerations should be given to a specific control program. Therefore, these results indicated that control methods are required to avoid loss due to milk fever. Milk fever is caused by a severe deficiency of metabolizable calcium ion in the circulation. This could be attributed to several risk factors. The risk factors identified in this study include milk yield, parity, and breed of the cows [28].

The prevalence rate of mixed disorders of 2.86% recorded in this study agrees with 1.05% reported by Simiret [26] and 1.03% reported by Haile et al. [8], but is lower than 5.6% indicated by Gashaw et al. [23] and 10.6% by Getachew and Nibret [22]. This variation could be due to interrelationship between reproductive problems as predisposing factors for each other.

## 5. Conclusion and Recommendations

Healthy cows are critical to maintain optimum reproductive performance. Reproductive efficiency is a critical component of dairy operation, whereas reproductive inefficiency is one of the most costly problems facing dairy industry today. The ultimate goal of dairy production is to lower the calving interval, increase herd productivity by decreasing number of services per conception, and increase profitability. But due to the reproductive problems encountered, the reproductive performance of dairy cattle was severely affected. So, the present study aimed to know the major reproductive disorders of dairy cattle and its associated risk factors in the study area. This study revealed that clinical mastitis, RB, RFM, vaginal and uterine prolapse, and abortion were the reproductive disorders with high prevalence, whereas dystocia, anestrus, hypocalcemia, uterine discharge, and stillbirth were recorded as RDs with lower prevalence. The biological and economic productivity of livestock production is highly influenced by reproductive performance. In order to improve the reproductive performance, understanding the reproductive disorders has been considered as practical solution.

Based on the above conclusion, the following recommendations are forwarded:

- Training the dairy owners on health education, feeding, and accurate heat detection to reduce the associated reproductive wastage
- Improvement in management system, proper selection of bull and appropriate timing of AI for breeding system, balanced feeding, and hygienic condition should be corrected
- Routine and periodical examination of cows postpartum and prepartum was essential
- Detailed studies were needed to identify etiology, distribution, and prevalence. So, the reproductive disorders in the study site were multifactorial

## Figures and Tables

**Table 1:** Socioeconomic characteristics of the respondents.

Parameter	Frequency	Percent
Sex of respondents		
Male	221	57.55
Female	163	42.45

Age of respondents		
10–20	0	0.00
21–30	113	29.43
31–40	181	47.14
41–50	76	19.79
>51	14	3.65

Level of education		
Illiterate	134	34.8
Elementary	119	31.07
Secondary	61	15.93
Diploma and above	70	18.28

Major farming activity		
Livestock production	125	32.55
Mixed crop-livestock production	259	67.45

System of production		
Intensive	96	25.0
Semi-intensive	210	54.69
Extensive	78	20.31

Composition of cattle herd		
Small	264	68.75
Medium	100	26.04
Large	20	5.21

Source of income		
Livestock	54	14.06
Crop and livestock	267	69.53
Salary	42	10.94
Other business	21	5.47

**Table 2:** Animal husbandry activities of the respondents.

Parameter	Frequency	Percent
Breed		
Local	262	68.23
Cross	122	31.77
Age of animal		
<3 years	52	13.54
3–5 years	58	15.10
5–7 years	128	33.33
>7 years	146	38.02
Parity		
Primiparous	114	29.68
Pluriparous	270	70.31
Mating system		
AI	158	41.15
NS	99	25.78
Both	127	33.07
Body condition		
Poor	179	46.61
Medium	174	44.53
Good	34	8.85

**Table 3:** The overall prevalence of reproductive disorders in dairy cattle.

Status of animal	Frequency	Percent
Cows with RDs	254	66.15
Cows without RDs	130	33.85

Total	384	100.00

**Table 4:** Prevalence of major reproductive disorders encountered in the study area.

Types of disorders	Frequency	Percent
Dystocia	15	3.91
Abortion	16	4.17
Clinical mastitis	79	20.57
Hypocalcemia	5	1.30
RFM	25	6.51
RB	68	17.71
Anestrus	7	1.82
Uterine and vaginal prolapse	21	5.47
Uterine discharge	4	1.04
Stillbirth	3	0.78
Mixed disorders	11	2.86

Total	254	66.15

**Table 5:** Associated risk factors with reproductive health problems of dairy cattle.

Factors	Variables	Total no. of cows examined	Total no. of cows affected	Percent	*χ*2	*p* value
Breed	Local	262	178	67.93	1.184	0.277
	Cross	122	76	62.29		

Parity	Primiparous	114	81	71.05	1.743	0.187
	Pluriparous	270	173	64.07		

BCS	Poor	61	42	68.85		
	Medium	260	171	65.76	32.91	0.000
	Good	63	41	65.07		

Age of animal	<3 years	52	34	65.38		
	3–5 years	58	40	68.98	13.23	0.004
	5–7 years	128	70	54.68		
	>7 years	146	110	75.34		

Production system	Intensive	96	62	64.58		
	Semi-intensive	210	121	57.61	28.484	0.000
	Extensive	78	71	91.02		

Hygiene	Poor	179	125	69.83		
	Medium	171	111	64.91	3.850	0.146
	Good	34	18	52.94		

Mating system	AI	158	100	63.29		
	NS	99	80	80.8	13.599	0.001
	Both	127	74	58.26		

**Table 6:** The association of prevalence rate of major reproductive problems with breed of the cows.

Type of disorder	Local (*n* = 262)		Cross (*n* = 122)		Level of significance
	F	%	F	%	
Dystocia	10	3.82	5	4.10	SNS
Abortion	11	4.20	5	4.10	SNS
PP mastitis	52	19.85	27	22.13	SNS
Hypocalcemia	1	0.38	4	3.28	SS
RFM	21	8.02	4	3.28	SNS
RB	45	17.18	23	18.85	SNS
Anoestrus	5	1.91	2	1.64	SNS
Uterine and vaginal prolapse	17	6.49	4	3.28	SNS
Uterine discharge	2	0.76	2	1.64	SNS
Stillbirth	3	1.15	0	—	SNS
Mixed disorder	11	4.20	0	—	SS

Total	178	67.93	76	62.29	SNS

SNS = statistically not significant (*p*-value >0.05), SS = statistically significant (*p*-value <0.05), *n* = number of observations, and *F* = frequency of observation.

**Table 7:** The association of prevalence rate of major reproductive problems with parity of the cows.

Type of disorder	Primiparous (*n* = 114)		Pluriparous (*n* = 270)		Level of significance
	F	%	F	%	
Dystocia	10	8.77	5	1.85	SS
Abortion	4	3.51	12	4.44	SNS
Mastitis	18	15.79	61	22.59	SNS
Hypocalcemia	0	—	5	1.85	SNS
RFM	12	10.53	13	4.81	SS
RB	21	18.42	47	17.41	SNS
Anoestrus	3	2.63	4	1.48	SNS
Uterine and vaginal prolapse	9	7.89	12	4.44	SNS
Uterine discharge	0	—	4	1.48	SNS
Stillbirth	1	0.88	2	0.74	SNS
Mixed disorder	3	2.63	8	2.96	SNS

Total	81	71.05	173	64.07	SNS

SNS = statistically not significant (*p*-value >0.05), SS = statistically significant (*p*-value <0.05), *n* = number of observations, and *F* = frequency of observation.

**Table 8:** The association of prevalence rate of major reproductive problems with body condition of the cows.

Type of disorder	Poor (n = 61)		Medium(n = 260)		Good (n = 63)		Level of significance
	F	%	F	%	F	%	
Dystocia	7	11.48	7	2.69	1	1.59	SS
Abortion	2	3.28	12	4.62	2	3.17	SNS
Mastitis	12	19.67	51	19.62	16	25.40	SNS
Hypocalcemia	0	—	2	0.77	3	4.76	SS
RFM	4	6.56	19	7.31	2	3.17	SNS
RB	10	16.39	47	18.08	11	17.46	SNS
Anoestrus	1	1.64	5	1.92	1	1.59	SNS
Uterine and vaginal prolapse	3	4.92	16	6.15	2	3.17	SNS
Uterine discharge	3	4.92	1	0.38	0	—	SS
Stillbirth	0	—	2	0.77	1	1.59	SNS
Mixed disorder	0	—	9	3.46	2	3.17	SNS

Total	42	68.85	171	65.76	41	65.07	SS

SNS = statistically not significant (*p*-value >0.05), SS = statistically significant (*p*-value <0.05), *n* = number of observations, and *F* = frequency of observation.

**Table 9:** The association of prevalence rate of major reproductive problems with age of the animals.

Type of disorder	<3 years (n = 52)		3–5 years (n = 58)		5–7 years (n = 128)		>7 years (n = 146)		Level of significance
	F	%	F	%	F	%	F	%	
Dystocia	6	11.54	4	6.90	0	—	5	3.42	SS
Abortion	0	—	2	3.45	2	1.56	12	8.22	SS
Mastitis	8	15.38	10	17.24	27	21.09	34	23.29	SNS
Hypocalcemia	0	—	0	-	0	—	5	3.42	SS
RFM	4	7.69	6	10.34	4	3.13	11	7.53	SNS
RB	11	21.15	11	18.97	29	22.66	17	11.64	SNS
Anoestrus	1	1.92	2	3.45	1	0.78	3	2.05	SNS
Uterine and vaginal prolapse	3	5.77	4	6.90	4	3.13	10	6.85	SS
Uterine discharge	0	—	0	—	1	0.78	3	2.05	SNS
Stillbirth	0	—	1	1.72	0	—	2	1.37	SNS
Mixed disorder	1	1.92	0		2	1.56	8	5.48	SNS

Total	34	65.38	40	68.96	70	54.68	110	75.34	SS

SNS = statistically not significant (*p*-value >0.05), SS = statistically significant (*p*-value <0.05), *n* = number of observations, and *F* = frequency of observation.

## Data Availability

The data used to support the findings of this study are included within the article and can be received from the author on request.
